# Variations in influenza vaccination coverage among the high-risk population in Sweden in 2003/4 and 2004/5: a population survey

**DOI:** 10.1186/1471-2458-7-113

**Published:** 2007-06-14

**Authors:** Madelon W Kroneman, Gerrit A van Essen

**Affiliations:** 1 NIVEL, Netherlands Institute of Health Services Research, P.O. Box 1568, 3500 BN Utrecht, The Netherlands; 2 European Scientific Working group on Influenza (ESWI), and Julius Center for Health Sciences and Primary Care, University Medical Center Utrecht, The Netherlands, P.O. Box 85060, 3508 AB Utrecht, The Netherlands

## Abstract

**Background:**

In Sweden, the vaccination campaign is the individual responsibility of the counties, which results in different arrangements. The aim of this study was to find out whether influenza vaccination coverage rates (VCRs) had increased between 2003/4 and 2004/5 among population at high risk and to find out the influence of personal preferences, demographic characteristics and health care system characteristics on VCRs.

**Methods:**

An average sample of 2500 persons was interviewed each season (2003/4 and 2004/5). The respondents were asked whether they had had an influenza vaccination, whether they suffered from chronic conditions and the reasons of non-vaccination. For every county the relevant health care system characteristics were collected via a questionnaire sent to the medical officers of communicable diseases.

**Results:**

No difference in VCR was found between the two seasons. Personal invitations strongly increased the chance of having had a vaccination. For the elderly, the number of different health care professionals in a region involved in administering vaccines decreased this chance.

**Conclusion:**

Sweden remained below the WHO-recommendations for population at high risk due to disease. To meet the 2010 WHO-recommendation further action may be necessary to increase vaccine uptake. Increasing the number of personal invitations and restricting the number of different administrators responsible for vaccination may be effective in increasing VCRs among the elderly.

## Background

Influenza continues to be a significant health risk for elderly persons and persons with chronic conditions (e.g. cardiovascular conditions, respiratory conditions, diabetes mellitus, renal failure, reduced resistance due to disease or treatment). All European countries have recommendations for influenza vaccination for these high risk groups [1]. WHO recommends that in 2006 50% of the high-risk population should be vaccinated, and that this rate should be increased to 75% in 2010. Despite relatively uniform recommendation policies, influenza vaccination coverage rates (VCRs) vary largely between different European countries [2,3]. In some countries (UK, Spain, The Netherlands) the 2010 target has already been reached for the elderly[4,5]. In some other countries the 2006 recommendation has been reached (Germany, Sweden). For the diseased, however, the situation is worse. In Germany, Poland, Sweden, and Spain, for instance, the 50% target has not yet been reached [4].

To increase the VCR, it is important to find out why uptake is low. Variation in VCR may be caused at three different levels. Firstly, personal preferences and characteristics of the potentially vaccinated persons may play a role. Secondly, different medical opinions of physicians that administer or recommend vaccinations may cause variation. Thirdly, the organisation of the health care system and especially the vaccination campaign may stimulate or frustrate vaccine uptake.

Ad 1 Personal reasons mentioned can be divided into beliefs and perceived barriers. Beliefs concern the perception of the severity of flu, perceived own susceptibility for influenza, ignorance of eligibility for the vaccination and forgetfulness [6-12]. Perceived barriers may be bad experiences in the past, practical problems (like timing of the appointment for vaccination and distance to administrator), financial barriers and (a negative) attitude towards vaccinations in general [4,7,13,14].

Ad 2 The role of the physician is quite important, since people that are informed about influenza vaccinations or receive a personal invitation of their physician are more likely to be vaccinated [15-21]. Research into the effect of reminders for physicians to immunize their patients was inconclusive [22,23].

Ad 3 Financial incentives for the physician may be the remuneration system. Here we assume that extra remuneration for influenza vaccinations will lead to higher uptake rates [2]. Research into the effect depending on which health professional administers the 'shot' is scarce. The British GP Willis, however, argues that this should happen in general practice [24]. The vast majority of studies into strategies to increase VCR has been conducted in General Practice settings [11,16,19,25-35], whereas few studies address interventions targeted on primary care nurses [36-38] or secondary care [38,39]. We assume that the chance of being vaccinated will increase if more different health professionals are involved, because of the wider choice and availability. Studies into VCR so far have addressed only one or two of the levels of variation. In this study we aim to combine all three levels.

Sweden did not yet reach the WHO-recommended vaccination uptake target for 2010. However, it is possible that under the current policy conditions, the VCR will increase gradually. In that case, we would expect an increase in VCR from the 2003/4 season to the 2004/5 season. This leads to our first research question:

• What is the development in VCR between the 2003/4 and the 2004/5 season in Sweden?

In Sweden the responsibility of the vaccination campaigns is delegated to the individual counties, which has resulted in different vaccination campaigns in the counties. The arrangements differ, for instance, on the following aspects: the health worker responsible for administering the vaccine (e.g., GP or public health worker), the way the administrators are remunerated (salaried or fee-for-service) and in out-of-pocket contributions for patients. This leads to our research question:

• What is the influence of personal preferences of the population, medical opinion of administrators and health care system characteristics on VCRs in Sweden?

## Methods

### Data collection

A population survey, by interviewing the respondents by phone, has been carried out in Sweden in April and May 2004 (2500 respondents) and in March and April 2005 (2500 respondents). The validated questionnaire was included in a regular omnibus [13], which is a large survey carried out on a regular basis and including different and changing subjects. Our questionnaire was included until the pre-defined number of respondents for our study were interviewed. As a result, the total length of the survey and the subjects included may change during the period that our questionnaire (consisting of 5 questions) was conducted. The questionnaires were administered by TNS, an international market information company, represented in Sweden by TNS Gallup. TNS subscribes to the ESOMAR/ICC code for market research. We choose this method because it is a relatively quick, efficient and low cost way to administer a short questionnaire like ours.

The respondents were aged between 15 and 74 years. Our questionnaire contained questions about vaccine uptake, self reported chronic conditions, reasons to refrain from vaccination, and whether one had received a personal invitation by a medical professional. The questions were defined; no open ended questions were included. For the self reported chronic conditions, we asked whether these were confirmed by a physician. Because the questionnaire was part of a larger omnibus, no response rates were available.

For every county we collected information about the remuneration-system of the administrators, the type of health worker involved in vaccination and the out-of-pocket payments for high-risk groups in 2004. A questionnaire was sent by email to the medical officers of communicable diseases (*Smittskyddsläkaren*) of every county. The response was 100%.

### Operationalisations

In this paper we use the term 'diseased' for people suffering from a chronic condition like cardiovascular conditions, respiratory conditions, diabetes mellitus, renal failure, reduced resistance due to disease or treatment, and who are younger than 65 years of age. In the questionnaire, examples were given to the respondents for cardiovascular and respiratory conditions ;the term used in the questionnaire for cardiovascular conditions was heart disease, with the following examples: 'chest pain, heart rhythm disorders, heart attack, or you have undergone heart surgery'; the term used for respiratory conditions was lung disease with the following examples: 'asthma, chronic bronchitis, pulmonary emphysema'; 'Elderly people' are 65 years of age and older; 'Healthy people' are below the age of 65 without a reported chronic condition.

Personal preferences were divided into misconceptions about, for instance, the risk of influenza, and barriers, causing people to have no vaccination despite their high risk condition. The demographic characteristics: household size and income were known for every respondent. The three largest disease groups (heart disease (yes/no), pulmonary disease (yes/no) and diabetes (yes/no)) were included in the analyses.

We did not have direct information on the medical opinion of, for instance, the GPs or other medical professionals about the high risk population in our sample. Thus, we assumed that health professionals that sent personal invitations to the high risk population had a more positive attitude towards vaccination compared to those who did not. Therefore, we asked the respondents whether they had had a personal invitation.

The regional health care system characteristics available in Sweden were: main administrator of the vaccination (dichotomised to the categories "GP" and "others"), number of different possible administrators (e.g. hospital specialists, public health officials, company physicians), payment system and extra payment for administrator for influenza vaccinations (either in salaried service or receiving fee-for-service) and out-of-pocket payments for the patients.

Ethical approval was not required for this study.

### Statistical analyses

For calculation of confidence intervals we used Fleiss quadratic 95% confidence intervals in the statistical package EpiInfo 6. In order to make the study population comparable with the real population, weight factors for region, age and gender were used that were provided by the organizer of the omnibus.

The influence of health care system characteristics were analysed using multilevel logistic analyses (MLwiN). For the diseased and the healthy group there was not enough variation between the counties to allow for this analysis. For those groups health care system characteristics were excluded from the analyses and ordinary logistic regression was used with the SPSS statistical package.

In the result section, when no differences between the two seasons could be demonstrated, we combined both seasons' data of the specific groups (e.g. results per region and reasons for non-vaccination among high-risk persons). Combining both seasons increased the number of observations, resulting in smaller confidence intervals.

## Results

### Representativeness of the sample

The age and gender distribution within the sample are comparable with the actual population, in the sense that the figures for the actual population fit within the confidence intervals of the sample (See Table 1). Compared to vaccines sales data [3] the VCR in our total sample is a little lower. The same is the case for the VCR of the elderly compared with a national Swedish study [40]. As far as we know, no data are available on the prevalence of the relevant chronic conditions under the age of 65 in the total population.

**Table 1:** Gender and age distribution and VCR in our sample compared to the actual population

		**This study**	**95%CI**	**Eurostat^1)^**
%female				
15–64 years old	2003	48.5	*46.4–50.6*	49.2
	2004	47.4	*45.2–49.5*	49.2
65–74 years old				
65–74 years old	2003	52.6	*46.9–58.1*	52.8
	2004	58.2	*52.2–63.7*	52.6
Total	2003	49.0	*47.1–51.0*	49.6
	2004	48.7	*46.6–50.6*	49.6
% 65–74 years old	2003	12.6	*11.3–14.0*	11.3
	2004	11.8	*10.6–13.2*	11.4
% vaccinated total population		**This study**		**MIV-group^2)^**
	2003	10.6	*9.8–12.3*	12.7
% vaccinated elderly		**This study**		**Sten^3)^**
	2003	45.6	*40–51*	51

1) Eurostat [52]; extraction date : 12-12-2006. The denominator for the percentages is the population aged 15–74 years.

2) Macroepidemiology of Influenza Vaccination (MIV) Study Group, 2005 [3].

3) Sten A., 2004 [40]

### VCR: variations between the seasons and the counties

About one out of four persons in our sample belongs to one of the high risk groups (elderly or diseased, see Table 2). Of the elderly, almost one out of two had been vaccinated in both seasons. For the diseased this was one out of 8. The VCR for the healthy persons was 4% in both seasons. The vaccination uptake did not differ significantly between the two seasons. Because there was no difference in age and gender distribution between the two seasons and no difference in VCR, we combined the data for the analysis of the differences between the counties. For the elderly the VCR ranged from less than 30% in Ostergotland, and Vasternorrland, Orebro and Jamtland to 78% in Jonkoping. For diseased persons, the VCRs ranged from 10% or less for Jonkoping, Skåne, Västra Götaland and Norbotten to 25% in Vasterbottens lan (See Table 3).

**Table 2:** Distribution of risk groups in the sample and vaccination coverage rate (VCR) per group in Sweden (after correction for age, sex and region)

**Season**		**Sample**		**Vaccinated persons**		
			
		**n**	**%^1)^**	**n**	**%^2)^**	***95% c.i.***
2003/2004	Elderly	316	13	144	46	*40–51*
	Diseased	332	13	43	13	*10–17*
	Healthy	1850	76	87	4	*4–6*
						
2004/2005	Elderly	296	12	133	45	*39–50*
	Diseased	313	12	38	12	*9–16*
	Healthy	1883	76	82	4	*3–5*

1) percentage of total sample

2) percentage of vaccinated persons within the risk group

**Table 3:** Overview of vaccination coverage rates in the Swedish counties in 2003/4 and 2004/5 combined and health care system characteristics concerning the influenza vaccination campaign, sorted by VCR for the elderly.

	Number of respondents and vaccination coverage							Vaccination campaign characteristics				
		
	Elderly		Diseased		Healthy		Total					
County	n	VCR (%)	N	VCR (%)	n	VCR (%)	n	Main adm.^1)^	Remuneration main administrator^2)^	No. of health care providers involved^3)^	Extra remuneration for administrator	Out of pocket payment elderly^4)^
Jonkoping	28	78	26	10	136	3	190	GP	Salary	1	no	no
Stockholm	93	65	97	16	691	6	881	GP	Salary	2	yes, each shot	no
Västmanland	23	64	16	0	109	5	148	GP	Capitation	3	yes, each shot	no
Sodermanland	15	48	20	12	127	2	162	Other^6)^	Salary	1	no	total
Gävleborg	18	48	28	13	129	2	175	GP	Salary	1	yes, each shot	no
Skåne	83	47	91	10	468	4	642	GP	Salary	4	yes, each shot	total
Blekinge	8	46	7	0	57	4	71	GP	Salary	3	no	no
Varmland	18	46	33	17	113	4	164	GP	Salary	4	no	no
Halland	18	43	16	15	98	5	132	PHW	Ffs	5	yes, each shot	partly
Västra Götaland	100	43	98	9	659	4	856	GP^6)^	Salary	2	no	total
Norrbotten	17	42	20	10	105	3	142	GP	Salary	2	no	no
Uppsala	28	39	31	17	190	8	248	GP	Salary	3	yes, each shot	total
Kalmar	21	36	16	16	95	1	131	GP	Salary	2	no	total
Vasterbottens lan	20	34	22	25	106	6	148	GP	Salary	3	no	total
Gotland	2	33	5	0	40	10	47	PHW	Salary	2	no	partly
Kronoberg	18	31	15	9	47	6	80	GP	Salary	4	yes, each shot	no
Dalarna	16	30	17	10	107	7	141	GP	Salary	4	no	no
Östergötland	36	27	38	15	177	5	251	GP	Salary	4	no	partly
Västernorrland	17	25	19	18	104	1	139	GP	Salary	4	no	total
Jamtland	15	24	15	16	43	2	73	PHW	Salary	4	yes, each shot	partly
Orebro	18	12	17	6	132	7	167	GP	Salary	2	no	total
Total	612		645		3733		4990					

1) Main administrator: GP = General Practitioner, PHW = Public Health worker

2) ffs = fee-for-service, capitation = fixed allowance for each patient on list

3) Number of different health care providers involved in influenza vaccination

4) total = total vaccination paid by elderly themselves, partly = part of the costs is paid by the elderly themselves

5) In Sodermanland, both GP and district nurses are involved in influenza vaccination

6) In Västra Götaland the shot is provided by nurses in General Practice

### Personal preferences

People in the high risk groups that had not been vaccinated mentioned reasons that mainly concerned misconceptions about influenza. The most frequently mentioned reasons were the perception not to qualify for a vaccination and perceived resistance. There was no difference between the two seasons in reasons mentioned. Table 4 displays the reasons for the two years combined. Both elderly and diseased mention the same reasons, except for considering influenza as a non-serious illness. This misconception appeared to be spread more widely among the diseased than the elderly.

**Table 4:** Reasons for not having a vaccination for high-risk group members1)2)

	**Elderly (n = 335)**	**Due to disease (n = 563)**
	**%**	**%**
*Misconceptions*		
		
I do not qualify for influenza vaccination	**22**	**26**
I have sufficient resistance to flu	**29**	**25**
Influenza is not a serious illness	7	**13**
		
*Barriers*		
		
The vaccination is too expensive	3	2
It slipped my mind	6	6
I was unable to attend at the given time	1	2
I have had bad experiences with influenza vaccination in the past	9	5
On principle, I am against vaccination	6	5
The GP or public health worker was too far away for me	1	1
		
*Miscellaneous*		
		
My physician considered it unnecessary	2	3
Other	26	24
Don't know	3	4

1) Combined results for 2003/4 and 2004/5, all percentages higher than 10% are displayed bold;

2) Since more than one answer was possible, the percentages may add up to more than 100%.

### Personal characteristics

Personal characteristics appear to be associated with vaccination uptake. People that were 70 years or older, females and those living in larger households were associated with a higher likelihood of vaccination.

In the diseased group, being female and being older than 50 years of age was associated with a higher chance of vaccination. There was no difference between the different diseases in the likelihood of vaccination. Of the people that were apparently healthy, those aged 50+ were more likely to be vaccinated.

### Invitations from health professionals

Personal invitations result in higher vaccination uptakes. 15% (95%-CI 13–17%) of the high risk population received a personal invitation. From those who did receive a personal invitation, 56% (95%-CI 49–63%) had been vaccinated. From those who did not receive such invitation, only 23% had been vaccinated (95% CI 21–26%). There was no difference between the two seasons.

### The effect of health care system characteristics

In most counties in Sweden the vaccination is administered by the GP, only 3 counties mention the public health worker as main vaccinator and in Sodermanland both GP and district nurse are equally involved. In almost all counties, the vaccinators receive a salary. Only two of the smaller counties (Halland and Västmanland) mentioned other remuneration systems. The number of different health care professionals involved varies from five different professionals in Halland (GP, public health worker, company physician, institutional physician and hospital based physician) to one in Gävleborg. In seven of the 21 counties, the administrators receive extra payment for vaccination and in 13 of the 21 counties out-of-pocket payment (partly or total vaccination) is required from the elderly; the same is the case for the diseased, except in Skåne, where there is variation within the county. (See Table 3).

The effect of health care system characteristics on the elderly could be tested. The number of administrators was associated with a lower likelihood of vaccination (See Table 5). Financial incentives were not associated with the likelihood of vaccination, although extra remuneration for vaccinating elderly people was just on the edge of significance, suggesting a positive effect of remuneration for GPs on vaccination uptake. For the diseased and the healthy, no variation at regional level was existent.

**Table 5:** The effect of demographic and health care system, characteristics and personal invitations on having had a vaccination in Sweden: results of logistic regression

	**Elderly^1)^**		**Diseased^2)^**		**Healthy^2)^**	
	odds ratio	sign.	odds ratio	sign.	odds ratio	sign.
**Personal characteristics**						
						
Household size	**1.38**	**0.05**	0.94	0.62	0.99	0.93
gender (male = ref. cat.)	**1.55**	**0.02**	**1.42**	**0.00**	0.11	0.77
age50plus/age70plus^3)^	**2.25**	**0.00**	**3.50**	**0.00**	**2.90**	**0.00**
						
**Health professional opinion**						
personal invitation	**3.52**	**0.00**	**2.80**	**0.00**	**6.13**	**0.00**
						
**Health care system characteristics**						
out-of-pocket payment	0.64	0.09				
extra remuneration for administrator	1.52	0.06				
GP is main administrator	0.86	0.59				
number of different administrators	**0.77**	**0.02**				
Constant	0.73		0.06		0.03	

1) Analysed with MLwiN Logistic regression

2) Analysed with SPSS logistic regression because there was no systematic regional variation existent

3) For each risk group the age of the respondents was dichotomised into two groups. For the diseased and healthy the division was made at the age of 50, for the elderly at the age of 70. The reference category for all risk groups was the younger age group.

## Discussion

The VCR of the Swedish elderly has almost reached the WHO-target for 2006. No difference in VCR was found between the two seasons. Sweden remained far below the WHO-target for the high-risk population due to disease. The fact that no change was found may be reason for concern, since without increase, the target of 2010 will certainly not be met.

High-risk persons refraining from vaccination were mainly guided by misconceptions. These misconceptions have increased in importance over the seasons. Perceived resistance and perceived non-qualification may be solved by information campaigns. Receiving a personal invitation for a vaccination remains an important way to increase the VCR which confirms already existing research evidence [11,16,25,27,30,34,41-43].

The different health care arrangements in the counties appeared to contribute, to some extent, to differences in VCR for the elderly. In counties where the administrator received extra remuneration, the elderly appeared to be more likely to be vaccinated, although the relationship was not significant. Despite of little research on this subject, the existing papers addressing the subject suggested a positive effect[19,27,29]. Interestingly, a larger number of different health professionals that could administer influenza vaccinations led to lower VCRs. An explanation may be that when more professionals are involved, none of them will feel responsible for the vaccination. There was no evidence that elderly were vaccinated less often in regions where out-of-pocket payments were requested, which is surprising in the light of other research findings suggesting that making influenza vaccination free of charge will increase the VCR [34,38].

The pattern found in Germany [4] and the Netherlands [13] that persons with cardiovascular disease and diabetes were vaccinated more often compared to those with pulmonary disease was not valid for Sweden. This may be due to the low VCR for the diseased in Sweden compared to Germany and the Netherlands.

Of course, this study has its limitations. The data on vaccination uptake and chronic conditions are based on self-reported data by the respondents. Although research into self-reported data compared to data from medical records revealed a satisfactory reliability of the self reported data, over-reporting as well as under-reporting could occur [4,13,44-48]. In Sweden in both years, 0.15 chronic condition per person was reported in the total sample. A second limitation concerned the fact that interviews were held in March and April. It would have been better to have a shorter interval between the vaccination period and the survey. However, in both years, the data collection took place in the same period, therefore, no systematic variation between the two years will occur due to different periods of data collection. A third limitation concerns the small number of respondents per county. This resulted in relatively large confidence intervals. However, due to practical limitations, a larger sample size was not possible.

Our study did not address the practical organisation of the vaccination campaign within GPs' offices. For instance, according to the literature, vaccination uptake may increase by offering vaccination clinics, offer influenza vaccination at every GP visit in the vaccination season, independent of the reason of the visit, and administer vaccines under standing orders (e.g. by practice nurses) [35,37-39,49-51]. However, the design of our study did not include practices as unit of observation and as a result we were not able to include this issue.

## Conclusion

The most important finding of this study is the lack of increase in VCR, which indicates that Sweden may not be able to meet the 2010 WHO-recommendation if no further action is undertaken concerning vaccine uptake. Personal invitations highly increase the chance of being vaccinated, so it seems to be important to persuade and facilitate administrators to invite those at risk. For the elderly, restricting the number of different professionals involved in vaccination may influence the VCR positively. Financial disincentives for the elderly themselves (out-of-pocket payments) did not influence the VCR. Personal characteristics (being older and female) were associated with higher VCRs. These characteristics cannot be influenced, but the results may instigate to target information campaigns towards the younger and male persons at risk.

## Competing interests

Financial competing interests

This study was financed by the European Scientific Working group on Influenza (ESWI).

Non-financial competing interests

The author(s) declare that they have no competing interests.

## Authors' contributions

MK participated in the design of the study, was responsible for the data collection and statistical analyses and drafted the manuscript. GVE conceived of the study and helped to draft the manuscript. Both authors read and approved the final manuscript.

## Pre-publication history

The pre-publication history for this paper can be accessed here:


